# Sarcoidosis or Tuberculosis: Should Corticosteroids Be Used?

**DOI:** 10.7759/cureus.47191

**Published:** 2023-10-17

**Authors:** Yuting Shen, Caihong Liu, Lan Zheng, Yuliang Zhao

**Affiliations:** 1 Department of Nephrology, Chengdu Seventh People’s Hospital (Affiliated Cancer Hospital of Chengdu Medical College), Chengdu, CHN; 2 Department of Nephrology, Kidney Research Institute, West China Hospital of Sichuan University, Chengdu, CHN; 3 Department of Nephrology, Zhongjiang County People’s Hospital, Deyang, CHN

**Keywords:** tuberculosis, sarcoidosis, interferon-gamma release assays, immune responses, corticosteroids

## Abstract

Sarcoidosis shows high similarity with tuberculosis in clinical manifestations and imaging features. It is rarely reported whether sarcoidosis patients with suspected latent tuberculosis can be treated safely with immunosuppressive therapy.

We reported on a 54-year-old man who presented with enlarged lymph nodes persisting for decades, accompanied by renal impairment and refractory hypercalcemia. The patient was diagnosed with sarcoidosis and suspected latent tuberculosis (as suggested by a positive tuberculin test and tuberculosis interferon-gamma release assays) and received prednisone under follow-up. The patient showed significant amelioration in hypercalcemia and shrinkage of lymph nodes, without evidence of developing active tuberculosis.

For sarcoidosis patients with suspected latent tuberculosis, immunosuppressive agents can be utilized safely based on close monitoring. Further efforts are required to reveal whether sarcoidosis and tuberculosis can trigger similar immune responses and what the clinical implications are.

## Introduction

Sarcoidosis is a multisystem disease with unknown etiology. More than 90% of sarcoidosis patients have lesions in the lungs that mainly present as pulmonary nodules and enlarged hilar and mediastinal lymph nodes [1]. Approximately 30% to 50% of patients have extrapulmonary manifestations [2], mainly occurring in the skin, peripheral lymph nodes, eyes, liver, and any other organs. The incidence of sarcoidosis varies by race, sex, and age, with a higher prevalence for women than for men in all ethnic groups. The incidence rate in black patients is double to triple that in white patients [3]. The etiology and pathogenesis of sarcoidosis are still not explicitly identified. Although some studies have shown that genetic susceptibility, immune state, environmental exposure, and other factors might jointly promote the occurrence of sarcoidosis [4], the exact pathogenic factors and pathogenic gene loci of the disease have not been found. Numerous studies have focused on the potential microbial etiology of sarcoidosis, most of which are *Mycobacteria* and *Propionibacterium* [5]. Although tuberculosis caused by *Mycobacterium tuberculosis* has a high degree of similarity to sarcoidosis in clinical manifestations, imaging features, and histopathological characteristics, in which epithelioid granuloma is the typical pathological change, the therapeutic strategies of the two diseases are quite different. Hence, differentiation between them is important for clinicians.

Here, we report a patient with sarcoidosis combined with suspected latent tuberculosis and discuss connections, differentiation approaches, and treatment strategies for the two diseases.

## Case presentation

A 54-year-old male patient was admitted due to an enlarged cervical lymph node persisting for 31 years, along with renal dysfunction and hypercalcemia for the past two years. Thirty-one years ago, an enlarged lymph node was discovered in the left neck, reaching its maximum size. It was reduced through perforation using unknown drugs but exhibited a recurrent feature. Fourteen years ago, he underwent a cervical lymph node biopsy, and *a benign node* was detected as recalled. Six years ago, pulmonary nodules and mediastinal nodules were also diagnosed. The patient has gradually lost approximately 16 kg of weight over the last decade. Two years ago, the patient was diagnosed with hypercalcemia and an elevation in serum creatinine (149 µmol/L) and was subsequently followed up by nephrologists. He denied a history of tuberculosis or contact with tuberculosis patients.

The patient had a height of 174 cm, weighed 58 kg, and had a body mass index (BMI) of 19.16 kg/m^2^. Upon admission, the patient showed normal vital signs and no fever. A subcutaneous mass, approximately 10 cm in diameter, was palpable on the left side of the neck, soft in texture, well-circumscribed, moderately mobile, and nontender. The suprasternal trachea deviated to the right.

**Figure 1:**
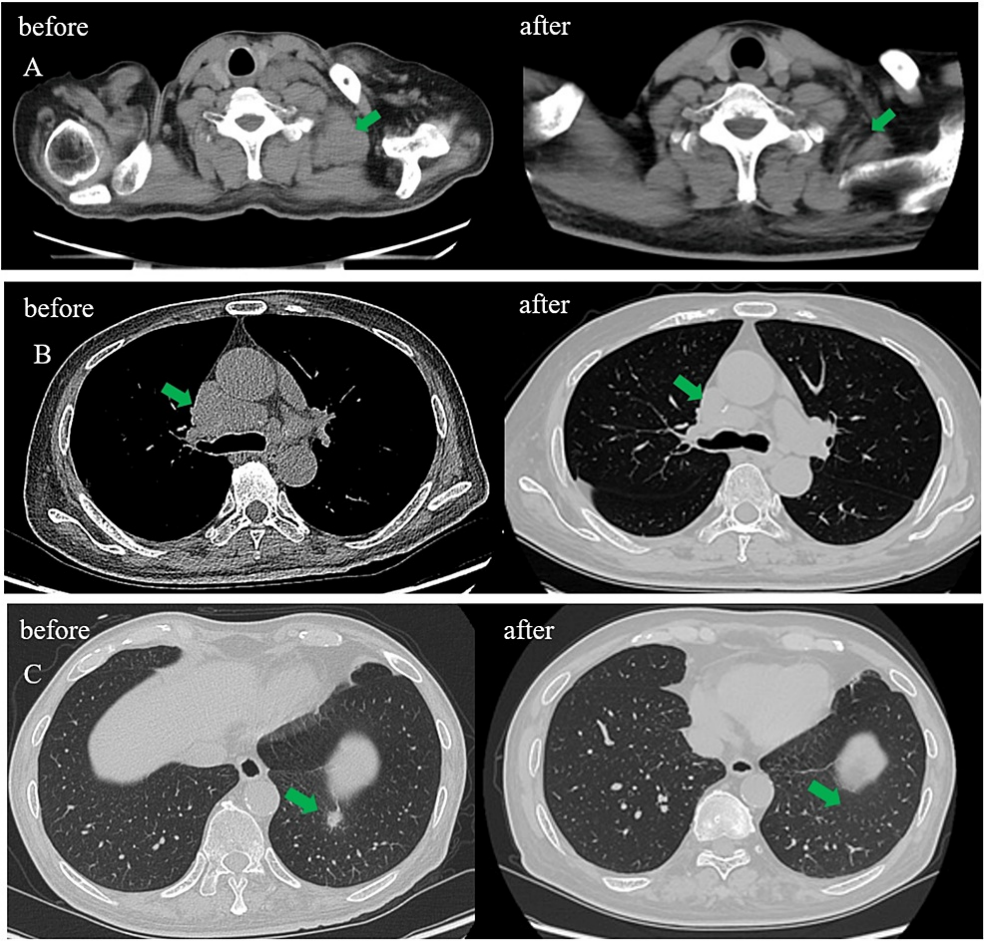
Panel A: Enlarged lymph nodes in the left cervical root on the chest CT scan (before), and lymph nodes diminished significantly after 18 weeks of prednisone acetate treatment (after); panel B: enlarged lymph nodes in the mediastinum on the chest CT scan (before), and lymph nodes diminished after 18 weeks of prednisone acetate treatment (after); panel C: enlarged nodules in the posterior basal segment of the lower lobe of the left lung on the chest CT scan (before), and nodules disappeared after 18 weeks of prednisone acetate treatment (after).

Lab tests were as follows: hemoglobin 101 g/L, serum creatinine 261 µmol/L, serum calcium 3.01 mmol/L, serum phosphorus 2.25 mmol/L, parathyroid hormone (PTH) 1.49 pmol/L, 24-hour urine calcium level normal, 25-VitD 29.8 nmol/L, adenosine deaminase (ADA) 30.65 IU/L, interleukin-2 receptor (IL-2R) 3,914 U/mL, angiotensin converting enzyme (ACE) 65 U/L, and β2-microglobulin 22.6 mg/L. Anti-nuclear antibody, anti-double-strand DNA antibody, anticardiolipin antibody, anti-neutrophil cytoplasmic antibody, and anti-glomerular basement membrane antibody were all negative. Urinalysis, tumor markers (including alpha-fetoprotein, carcinoembryonic antigen, neuron-specific enolase, squamous cell carcinoma antigen, and Cyfra21-1), C-reactive protein, and ferritin showed no abnormalities. Although the purified protein derivative (PPD) test was positive and the tuberculosis interferon-gamma release assay (TB-IGRA) test was 4941.39 pg/mL, the results from repeated sputum smear/culture and quantitative polymerase chain reaction (qPCR) for *M. tuberculosis* were all negative. A chest CT scan revealed multiple enlarged lymph nodes in the mediastinum and left cervical root, along with nodules in the posterior basal segment of the lower lobe of the left lung (Figures 1A-1C, the pictures are marked with the term *before*).

Ultrasound examination of the cervical lymph nodes indicated that the left cervical lymph nodes were enlarged, some of which were structurally abnormal. Bilateral renal calculi were also observed by urinary ultrasound.

After admission, the patient was hydrated and salmon calcitonin was administered to decrease the calcium level. Although serum calcium decreased transiently, it rebounded soon after salmon calcitonin withdrawal. The serum creatinine climbed from 239 to 292 µmol/L upon admission.

**Figure 2:**
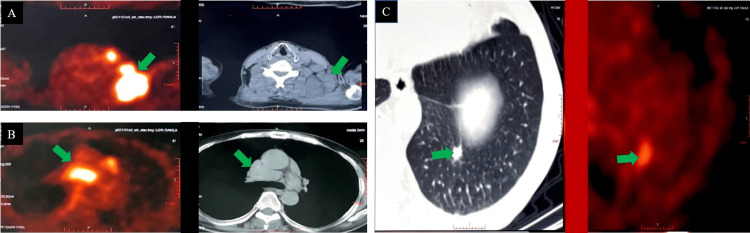
Panel A: PET-CT highlights area showed increased FDG uptake in the cervical lymph nodes; panel B: increased FDG uptake in the thoracic lymph nodes; panel C: increased FDG uptake in the posterior basal segment of the lower lobe of the left lung. PET-CT, positron emission tomography-computed tomography; FDG, fluorodeoxyglucose

A cervical lymph node biopsy was performed, revealing granulomatous inflammation without definite necrosis. IgG4-positive cells were present in less than 5 per high-power field (HPF), and results for acid-fast stain, periodic acid-Schiff stain, and hexamine silver were negative. In addition, tuberculosis DNA fragments were not detected by qPCR. Positron emission tomography-computed tomography (PET-CT) revealed abnormal elevation in glucose metabolism in the cervical and thoracic lymph nodes, as well as in the posterior basal segment of the lower lobe of the left lung (Figure 2).

According to the 2020 guidelines for the clinical diagnosis and monitoring of sarcoidosis proposed by the American Thoracic Society [6], the diagnosis of sarcoidosis is based on (1) clinical manifestations, (2) finding of no necrotizing granulomatous inflammation in one or more tissue samples, and (3) the exclusion of other causes of granulomatous diseases. The patients suffered enlarged cervical and thoracic lymph nodes for decades showing non-necrotizing granulomatous inflammation with high glucose metabolism, accompanied by increased serum calcium, ADA, IL-2R, ACE, and β2-microglobulin levels. Although being positive in the PPD test and TB-IGRA, they cannot be considered dependent diagnostic tools for tuberculosis, as the gold standard for tuberculosis is culture. Moreover, lymph node histopathology showed granulomatous inflammation without caseous necrosis.

**Figure 3:**
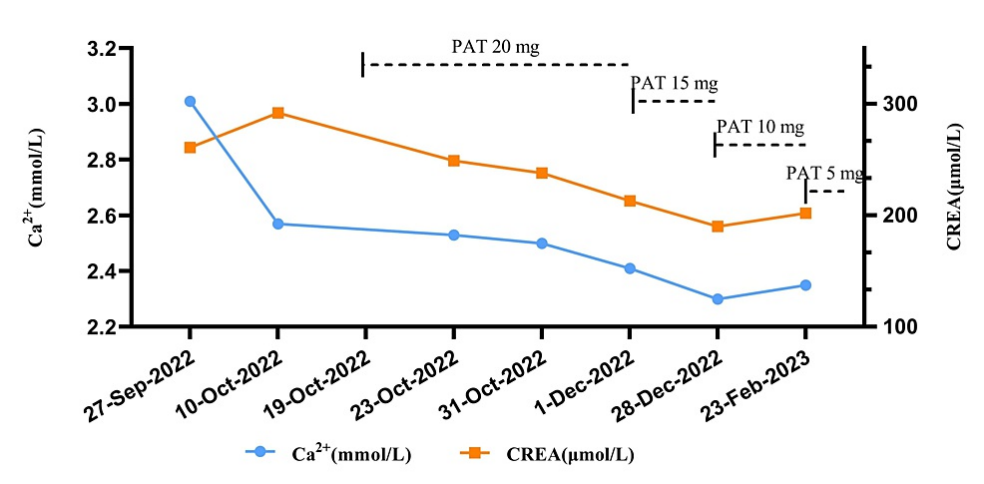
Trend in serum creatinine and Ca2+ levels for the patient over time. PAT, prednisone acetate tablets; CREA, serum creatinine; Ca2+, serum calcium

The patient was clinically diagnosed with sarcoidosis and given oral prednisone acetate tablets 20 mg once daily. The serum calcium stabilized at a normal range, and creatinine gradually decreased to a minimum of 190 µmol/L (Figure 3).

Symptoms of numbness and weakness of the limbs were also ameliorated. The patient was successfully discharged with regular follow-up. CT reexamination after four months of corticosteroid therapy illustrated that the cervical and mediastinal lymph nodes and pulmonary nodules were significantly diminished compared with those before treatment (Figures 1A-1C, the pictures are marked with the word *after*). Prednisone acetate tablets were tapered down to 5 mg once daily for maintenance treatment.

## Discussion

In this study, we reported a male patient with renal impairment and refractory hypercalcemia, accompanied by cervical lymphadenopathy and thoracic nodules for years. Two lymph node biopsies showed no evidence of tuberculosis or tumor, but the levels of ACE, ADA, and IL-2R were elevated. According to the 2020 American Thoracic Society guidelines for sarcoidosis [6], the patient was diagnosed with sarcoidosis. We gave the patient prednisone acetate tablets 20 mg once daily as per guidelines [2], and hypercalcemia was ameliorated. Chest CT after four months of corticosteroid therapy showed significant shrinkage of pulmonary/mediastinal/cervical lesions, thus further justifying our diagnosis of sarcoidosis.

The patient was positive in the PPD test and TB-IGRA, but negative in sputum smear/sputum culture or neck mass qPCR for *M. tuberculosis* DNA fragment. The specificity of the PPD test in the diagnosis of tuberculosis was unsatisfactory, which may be because the PPD test is greatly affected by BCG vaccination, the immune status of the patient, and the subjective interpretation of results. TB-IGRA can exclude the interference of BCG vaccination, so it is viewed as a test with higher sensitivity and specificity than the PPD test [7]. The positive PPD test and TB-IGRA result in this patient reminded us of the possibility of latent tuberculosis, which warranted close monitoring, especially in the setting of immunosuppressive therapy with corticosteroids.

Sarcoidosis and tuberculosis showed high similarity in clinical manifestations, imaging features, and pathological characteristics, with pulmonary nodules and lymph node enlargement clinically, elevated glucose metabolism in PET-CT, and granulomatous inflammation pathologically. The potential connection between these two diseases has been extensively studied. Some studies have suggested that granuloma formation in sarcoidosis is the result of excessive immune reactions to unknown antigens [8,9]; therefore, further exploration is required to reveal whether strongly positive PPD test and TB-IGRA are the result of sarcoidosis overreaction and whether their intensity is paralleled to sarcoidosis activity. Previous studies also showed that T-cells in sarcoidosis respond to various mycobacterial proteins [10-13], and monocytes in the blood of patients with sarcoidosis increase the production of interferon-γ upon stimulation with tuberculosis antigens, including ESAT6 and KatG [12]. An Indian study showed that the QuantiFERON-TB Gold In-Tube test was also positive in some patients with sarcoidosis, and there may be a common immune response pathway in patients with tuberculosis and sarcoidosis, which accounts for PPD positivity and elevated TB-IGRA [14]. Tuberculosis and sarcoidosis cannot be differentiated based on TB-IGRA positivity alone.

It is also generally believed that sarcoidosis is caused by the inflammatory reaction to environmental antigens in individuals with genetic susceptibility. *M. tuberculosis* antigen is one of the causes of sarcoidosis [15], in addition to *Propionibacterium acnes*, fungi, and pesticides. Different studies have observed similar pathogen components of *M. tuberculosis* in sarcoidosis tissues [8,9,16]. The gene expression signature of sarcoidosis also exhibited a highly similar pattern in *M. tuberculosis* infection, with shared proinflammatory and signaling pathways [17]. Furthermore, peripheral blood from patients with sarcoidosis and tuberculosis infection displayed widely overlapping transcriptional signatures [17,18]. Based on the aforementioned findings, some have proposed an interesting hypothesis that sarcoidosis and tuberculosis are two ends of the same disease spectrum [19], which has not yet been further confirmed.

## Conclusions

Here, we report a rare case of sarcoidosis with an exceptionally high level of TB-IGRA. Patients with sarcoidosis can be prone to misdiagnosis with tuberculosis, potentially resulting in delayed or inappropriate treatment. This case demonstrated that when sarcoidosis is complicated by suspected latent tuberculosis, immunosuppressive agents can be used safely based on close monitoring. Sarcoidosis and tuberculosis share similar clinical manifestations and are interconnected in terms of pathogenesis. The potential relationship between TB-IGRA and sarcoidosis, as well as tuberculosis, is elucidated in this case. Further efforts are required to reveal whether sarcoidosis and tuberculosis can trigger similar immune responses, such as those seen in the PPD test or IGRA, and what the clinical implications are.
